# Ultraviolet radiation accelerates NRas-mutant melanomagenesis: A cooperative effect blocked by sunscreen

**DOI:** 10.1111/pcmr.12601

**Published:** 2017-07-04

**Authors:** Rebecca C. Hennessey, Andrea M. Holderbaum, Anamaria Bonilla, Conor Delaney, James E. Gillahan, Kathleen L. Tober, Tatiana M. Oberyszyn, Jonathan H. Zippin, Christin E. Burd

**Affiliations:** 1 Department of Cancer Biology and Genetics, Biomedical Research Tower, The Ohio State University, Columbus, OH, USA; 2 Department of Molecular Genetics, Biomedical Research Tower, The Ohio State University, Columbus, OH, USA; 3 Department of Pathology, The Ohio State University, Columbus, OH, USA; 4 Department of Dermatology, Joan and Sanford I. Weill Medical College of Cornell University, New York, NY, USA

**Keywords:** melanoma, mouse model, NRAS, sunscreen, ultraviolet

## Abstract

To mitigate melanoma risk, sunscreen use is widely advocated; yet, the ability of sunscreens to prevent melanoma remains controversial. Here, we test the tenet that sunscreens limit melanoma risk by blocking ultraviolet radiation (UV)-induced DNA damage using murine models that recapitulate the genetics and spontaneous evolution of human melanoma. We find that a single, non-erythematous dose of UV dramatically accelerates melanoma onset and increases tumor multiplicity in mice carrying an endogenous, melanocyte-specific *NRas^61R^* allele. By contrast, transient UV exposure does not alter tumor onset in mice lacking p16^INK4a^ or harboring an *NRas^12D^* allele. To block the rapid onset of melanoma cooperatively caused by UV and *NRas^61R^*, we employed a variety of aerosol sunscreens. While all sunscreens delayed melanoma formation and blocked UV-induced DNA damage, differences in aerosol output (i.e., amount applied/cm^2^) caused variability in the cancer preventative efficacy of products with identical sunburn protection factor (SPF) ratings.

## 1 | INTRODUCTION

Sunscreen is intended to mitigate skin cancer risk by blocking the carcinogenic effects of ultraviolet radiation; yet, the ability of sunscreens to prevent the most deadly form of skin cancer, melanoma, is debated (Iannacone, Hughes, & Green, 2014; Maslin, 2014). Only a single randomized control trial has ever assessed the ability of sunscreens to prevent melanoma, and challenges associated with such work make the testing and comparison of individual sunscreen products impractical (Green, Williams, Logan, & Strutton, 2011; Green et al., 1999). Consequently, there are no premarket tests to assess how effectively a particular sunscreen product prevents melanoma. The only biological measurement used to rate commercial sunscreen efficacy is the sunburn protection factor (SPF), which indicates how well a sunscreen prevents ultraviolet B (UVB)-induced erythema and inflammation. This rating schema ignores experimental data suggesting that even non-burning sun exposures can promote melanoma initiation and is misleading to consumers who view sunscreens as proven cancer preventatives (Seite, Fourtanier, Moyal, & Young, 2010; Viros et al., 2014).

The rationale for measuring sunburn derives predominantly from the tenet that blocking UVB-induced DNA damage will prevent melanomagenesis. While the mutational burden of human melanomas can be extremely high (Cancer Genome Atlas Network, 2015), the relationship between UVB-induced DNA damage and tumor initiation is unclear. Common melanoma driver mutations (i.e., NRAS codon 61 and BRAF codon 600 variants) are not characterized by a UV signature and are found frequently in benign melanocytic nevi (i.e., moles), including those present at birth (Bauer, Curtin, Pinkel, & Bastian, 2007; Hodis et al., 2012; Pollock et al., 2003; Poynter et al., 2006; Ross, Sanchez, & Grichnik, 2011). Thus, melanoma precursor lesions are genetically primed for transformation. Prior studies implicate *Trp53* mutations and immune cell recruitment in the UV-mediated transformation of genetically primed murine melanocytes (Mukhopadhyay, Ferguson, Muller, Handoko, & Walker, 2016; Viros et al., 2014; Zaidi et al., 2011). However, these experimental systems do not simultaneously model both endogenous oncogene expression and the well-established epidemiological link between intermittent sun exposure and melanoma risk (Whiteman, Whiteman, & Green, 2001). This is of relevance because exogenous RAS mutants are known to mislocalize within the cell and hyperstimulate downstream signaling pathways to levels which are not seen upon endogenous gene activation (Omerovic & Prior, 2009; Pylayeva-Gupta, Grabocka, & Bar-Sagi, 2011).

We previously reported the development of a genetically engineered murine model, *TpN^61R^*, which faithfully recapitulates the somatic acquisition of oncogenic *NRAS* mutations observed in 61%–81% of congenital nevi and 10%–25% of cutaneous melanomas (Bauer et al., 2007; Burd et al., 2014; Cancer Genome Atlas Network, 2015; Forbes et al., 2015). As transient sun exposures are more strongly linked to melanoma risk than chronic sun exposures (Whiteman et al., 2001), we used derivatives of this model to examine how UV treatment influences spontaneous melanoma development. Capitalizing on the exquisite UV sensitivity of our clinically relevant system, we evaluated the melanoma preventative efficacy of a wide variety of sunscreen formulations in the hopes of providing scientific evidence to guide physician recommendations as well as insight into the processes that govern melanomagenesis.

## 2 | RESULTS

### 2.1 | A single, early-life UV exposure accelerates melanomagenesis in *TpN^61R^* mice

**FIGURE 1:**
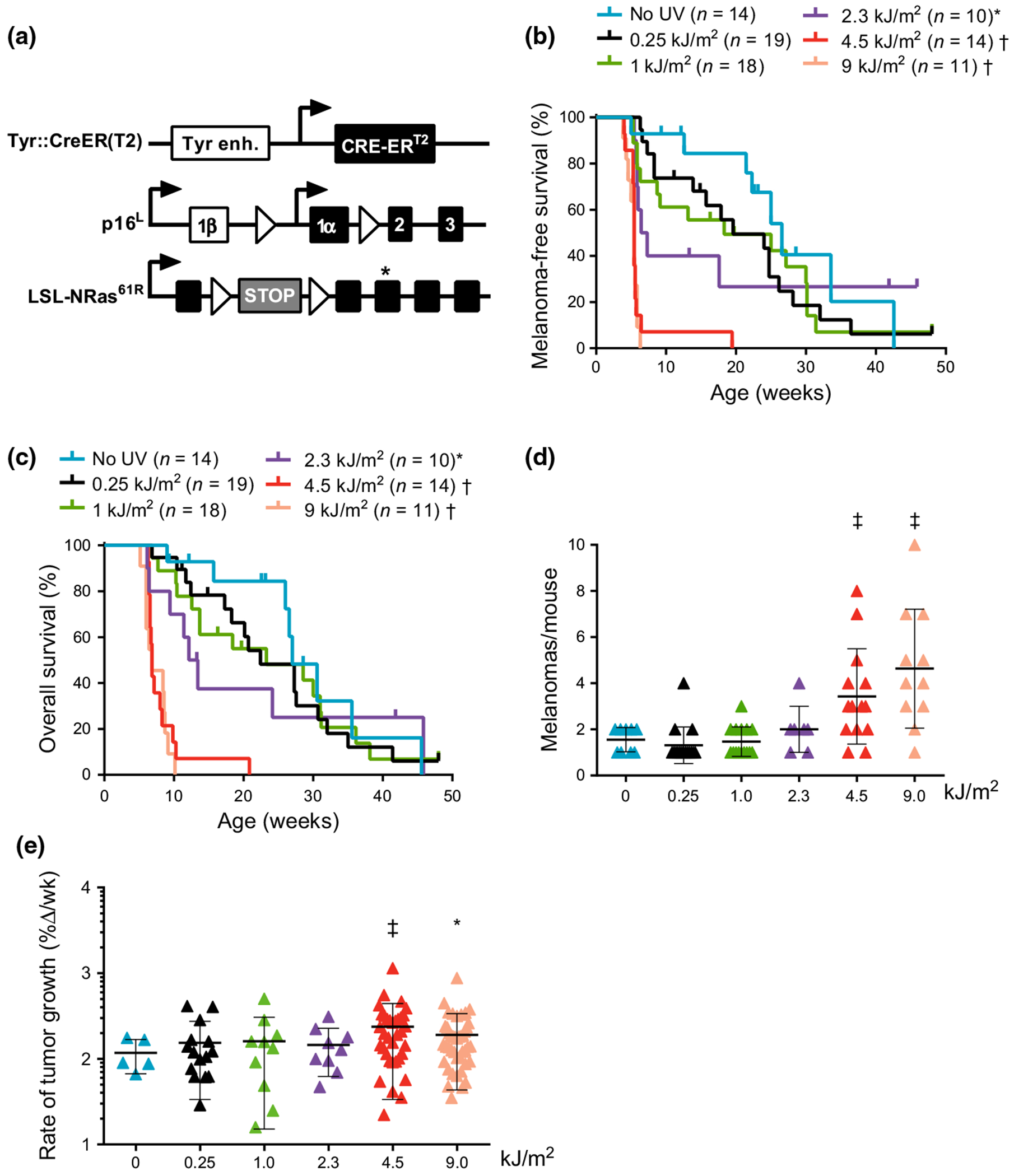
A single UV exposure decreases melanoma onset in a dosedependent manner. (a) *TpN^61R^* mice contain a melanocyte-specific, tamoxifen-i nducible CRE allele (*Tyr::CreER(T2)*), a conditional *p16* knockout allele (*p16^L^*), and a conditional oncogenic *NRas^61R^* allele (*LSL-NRAS^61R^*). Open triangles represent Lox P sites. (b) Melanoma-free survival of UV- and mock-exposed *TpN^61R^* mice. (c) Overall survival of UV- and mock-exposed *TpN^61R^* mice. (d) Tumor burden for UV- and mock-exposed *TpN^61R^* mice. Each triangle represents a single mouse. Data are mean ± standard deviation. (e) Average tumor growth rates for UV- and mock-exposed *TpN^61R^* mice. Each triangle represents a single tumor (0 kJ/m^2^, *n* = 5; 0.25 kJ/m^2^, *n* = 15; 1 kJ/m^2^, *n* = 10; 2.3 kJ/m^2^, *n* = 9; 4.5 kJ/m^2^, *n* = 35; 9 kJ/m^2^, *n* = 43). Data are mean ± standard deviation. *p ≤ .05, ^‡^*p* ≤ .01, ^†^*p* ≤ .0001 compared to No UV control [Colour figure can be viewed at wileyonlinelibrary.com]

The *TpN^61R^* model harbors clinically relevant, melanocyte-specific somatic gene alterations in *NRas* and *Cdkn2a* (*p16^INK4a^*) (Figure 1a). Specifically, *NRAS* codon 61 mutations are observed in human congenital nevi (61%–81%), blue nevi (3%), intradermal nevi (8%), and metastatic melanomas (10%–25%), whereas p16^INK4a^ loss of function occurs in ~69% of melanomas (Bauer et al., 2007; Cancer Genome Atlas Network, 2015; Forbes et al., 2015; Shain et al., 2015). To determine how transient UV exposure influences melanomagenesis in these predisposed animals, we treated three-day-old *TpN^61R^* pups with a single, biologically relevant dose of UVB-centered light (See Methods). After treatment, no visual or histological evidence of edema, erythema, or blistering was seen at any administered UV dose (data not shown). Early-l ife UV exposure caused a dose-dependent decrease in the melanoma-free survival (MFS) of *TpN^61R^* mice that was significant at doses greater than or equal to 2.3 kJ/m^2^, but never exceeded the 80% decrease observed at 4.5 kJ/m^2^ (Figure 1b; *p* = .032, 2.3 kJ/m^2^; *p* < .0001 for doses ≥4.5 kJ/m^2^). Overall survival (OS) also decreased in UV-treated *TpN^61R^* animals (Figure 1c; *p* = .031, 2.3 kJ/ m^2^; *p* < .0001 for doses ≥4.5 kJ/m^2^). In mice treated with a single dose of 0.25, 1.0 or 2.3 kJ/m^2^ UV, early mortality was attributed to rapid tumor onset rather than increased tumor burden, growth rate, or ulceration (Figure 1d,e and data not shown). By contrast, increases in melanoma burden and growth rate contributed to earlier euthanasia in *TpN^61R^* mice treated with 4.5 or 9 kJ/m^2^ UVB (Figure 1d,e).

### 2.2 | *NRas^61R^* cooperates with UV to drive melanoma initiation

**FIGURE 2:**
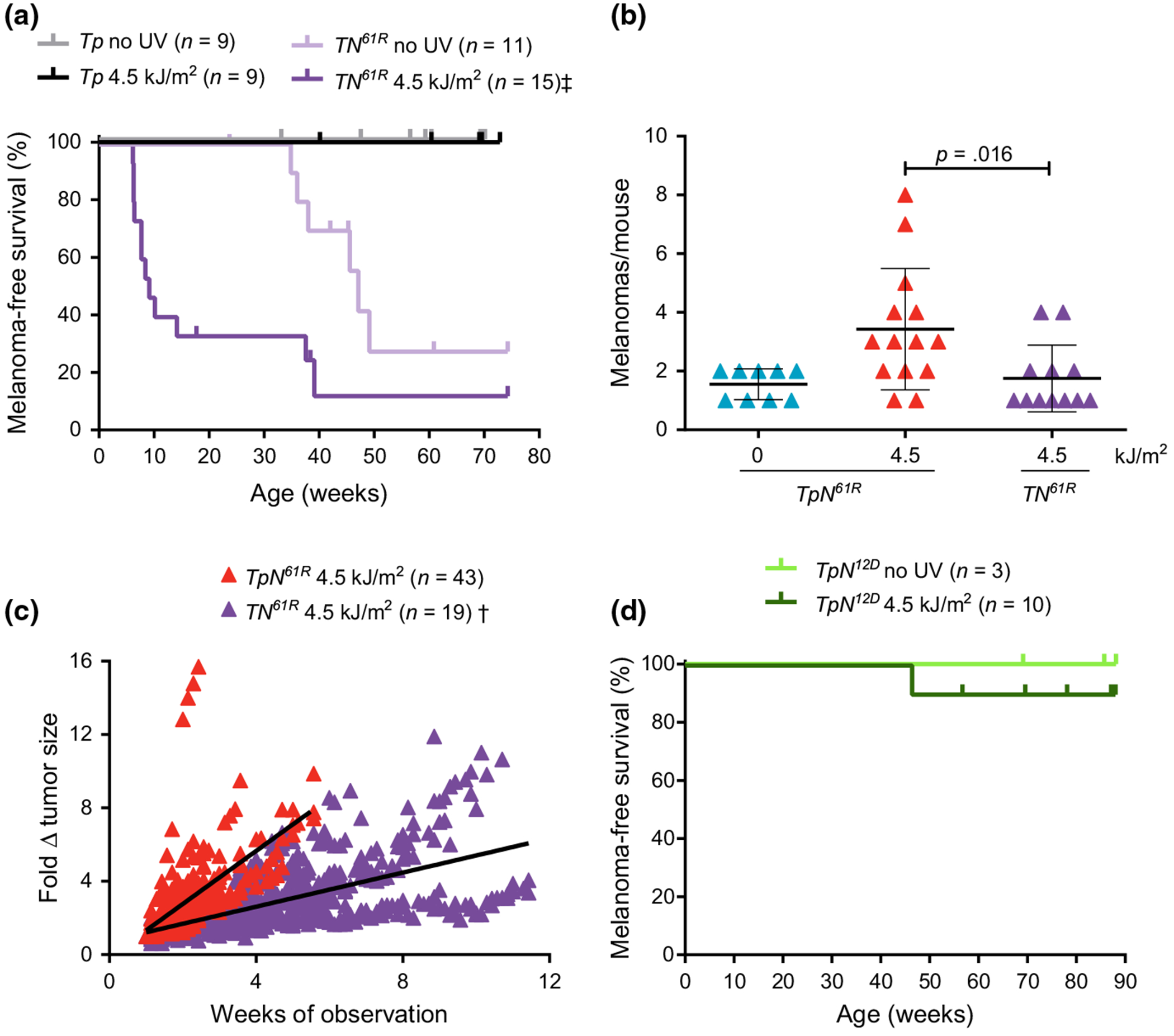
UV cooperates with NRas codon 61 mutants to drive melanoma. (a) Melanoma-free survival of UV- and mock-exposed *Tp* and *TN^61R^* mice. (b) Final tumor burden in UV- and mock-exposed *TpN^61R^* and *TN^61R^* mice. Each triangle represents a single mouse (*TpN^61R^* 0 kJ/m^2^, *n* = 9; *TpN^61R^* 4.5 kJ/m^2^, *n* = 14; *TN^61R^* 4.5 kJ/m^2^, *n* = 12). Data are mean ± standard deviation. (c) Measured change in melanoma size over time in UV-induced *TpN^61R^* (red; *n* = 43 tumors) and *TN^61R^* (purple; *n* = 19 tumors) tumors. Tumors were measured five times weekly from discovery until reaching the pre-established exclusion criteria. Each triangle indicates change in growth for a single tumor at that time point. (d) Melanoma-free survival of UV- and mock-exposed *TpN^12D^* mice. ^‡^*p* ≤ .01, ^†^*p* ≤ .0001 [Colour figure can be viewed at wileyonlinelibrary.com]

*NRAS* mutations are UVB-independent genetic events that occur early in the evolution of melanoma, whereas p16^INK4a^ loss is often associated with malignant progression (Shain et al., 2015). Using inbred variations of the *TpN^61R^* model, we sought to determine which genetic lesion, *NRas^61R^* or *p16^INK4a^* loss, was the major determinant of UV-induced tumor susceptibility. *Tp* mice, which lack melanocytic p16^INK4a^ expression, did not develop melanoma after a single exposure to 4.5 kJ/m^2^ UV (Figure 2a). However, one dose of UV cooperated with NRas^61R^ alone to drive melanoma formation 80% faster than in untreated controls (Figure 2a, *p* = .002). While *TN^61R^* mice were sensitive to UV-mediated melanoma initiation, these mice developed fewer tumors than the UV-treated *TpN^61R^* model, indicating that p16^INK4a^ loss facilitates transformation (Figure 2b, *p* = .016). *TN^61R^* melanomas also grew more slowly than *TpN^61R^* tumors (Figure 2c, *p* < .0001) and showed a slight delay in onset (Median MFS *TN^61R^* = 9.1 weeks, *TpN^61R^* = 5.4 weeks; *p* < .0001). Overall, the acceleration of melanoma formation by UV was similar in both the *TN^61R^* and *TpN^61R^* models (80.6% and 79.6% faster, respectively), suggesting that NRas activation, rather than p16^INK4a^ loss, is the major cooperating factor in sunlight-induced melanoma. However, further analyses revealed that *TN^61R^* melanomas frequently show evidence of either p16^INK4a^ loss (1/11 tumors) or p53 stabilization (7/17 tumors) (Figure S1), thus implicating loss of the *Cdkn2a* locus in tumor progression.

In human melanoma, oncogenic *NRAS* mutations predominately localize to codon 61 (Cancer Genome Atlas Network, 2015; Forbes et al., 2015). By contrast, oncogenic *NRAS* variants localizing to codons 12 and 13 are more prevalent in acute myeloid leukemia and colorectal carcinoma (Forbes et al., 2015). We examined whether endogenous, melanocyte-specific NRas^12D^ expression would also cooperate with a single dose of UV to promote melanoma onset. After 80 weeks of observation, only one melanoma was observed in ten UV-exposed *TpN^12D^* mice (Figure 2d).

### 2.3 | Sunscreens block UV-induced DNA damage and delay melanoma formation

Sunscreens are sold in a variety of product formulations (i.e., sprays, sticks, balms, lotions, and gels) and contain distinct combinations of active (e.g., benzophenones, cinnamates, salicylates, zinc oxide, titanium dioxide) and inactive (e.g., vitamins, fragrances, preservatives) ingredients. In this study, we tested aerosol sunscreens as they can be evenly applied and dry quickly on non-anesthetized animals. Six aerosol sunscreens with varying SPF, chemical, mineral, and bioactive excipient compositions were selected for examination (Table 1). Sunscreens were applied to *TpN^61R^* mice and allowed to dry prior to the administration of a single 4.5 kJ/m^2^ dose of UV. All sunscreens, regardless of composition or SPF rating, extended the MFS and OS of UV-exposed *TpN^61R^* mice (Table 1). Additionally, significant decreases in melanoma burden were observed after the application of all sunscreens except sunscreens three and six (Table 1).

### 2.4 | DNA damage prevention does not determine sunscreen efficacy

**FIGURE 3:**
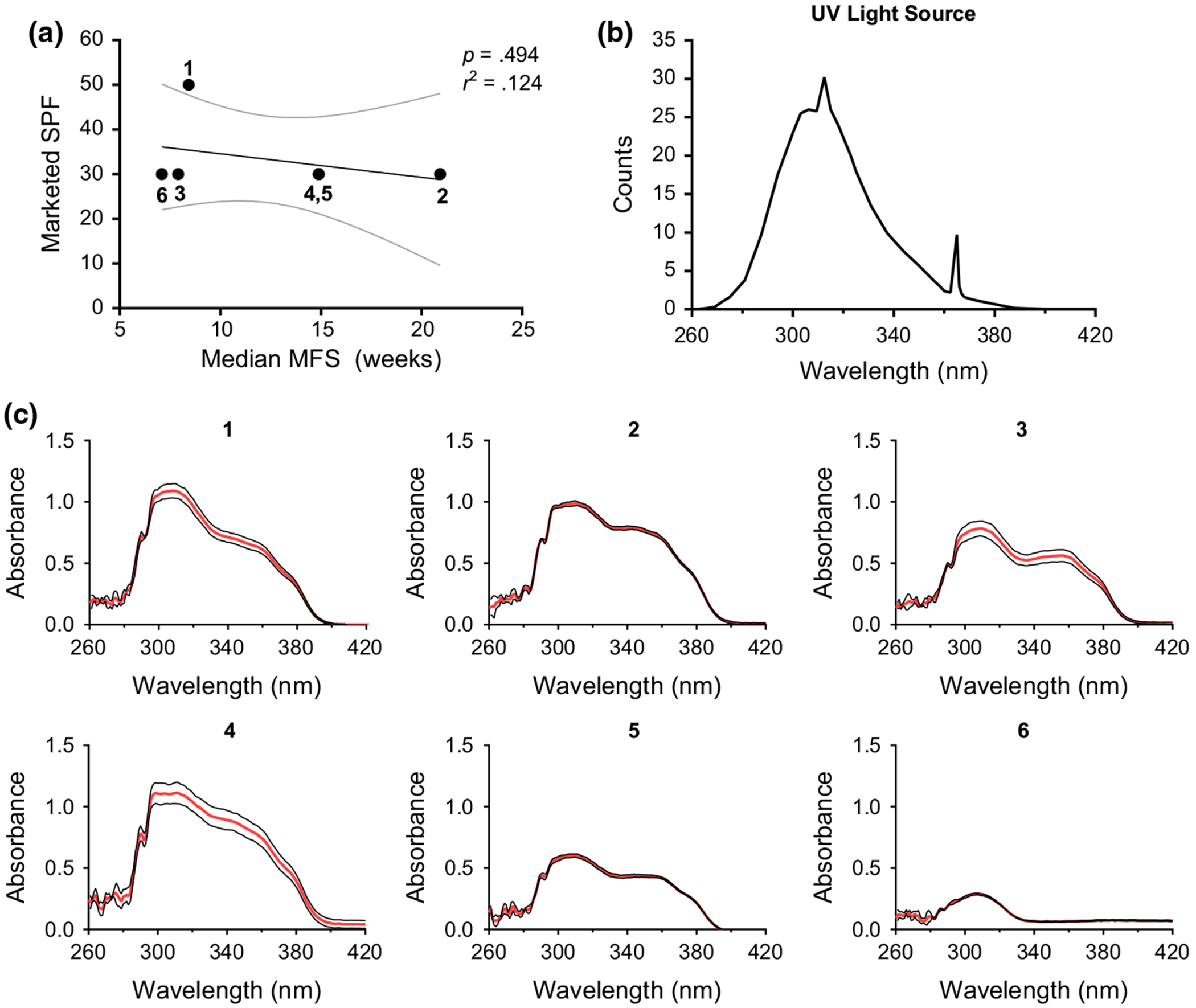
SPF and spectral coverage do not explain differences in sunscreen efficacy. (a) Linear regression of marketed SPF versus median melanoma-free survival (see Table 1) for each sunscreen product. Numbers correspond to individual sunscreen products. 95% confidence intervals are shown in gray. (b) Spectral output of the experimental light source. (c) Average UV absorbance of commercial sunscreens (red) tested on three separate occasions with 95% confidence intervals (black). Numbers correspond to individual sunscreen products [Colour figure can be viewed at wileyonlinelibrary.com]

**FIGURE 4:**
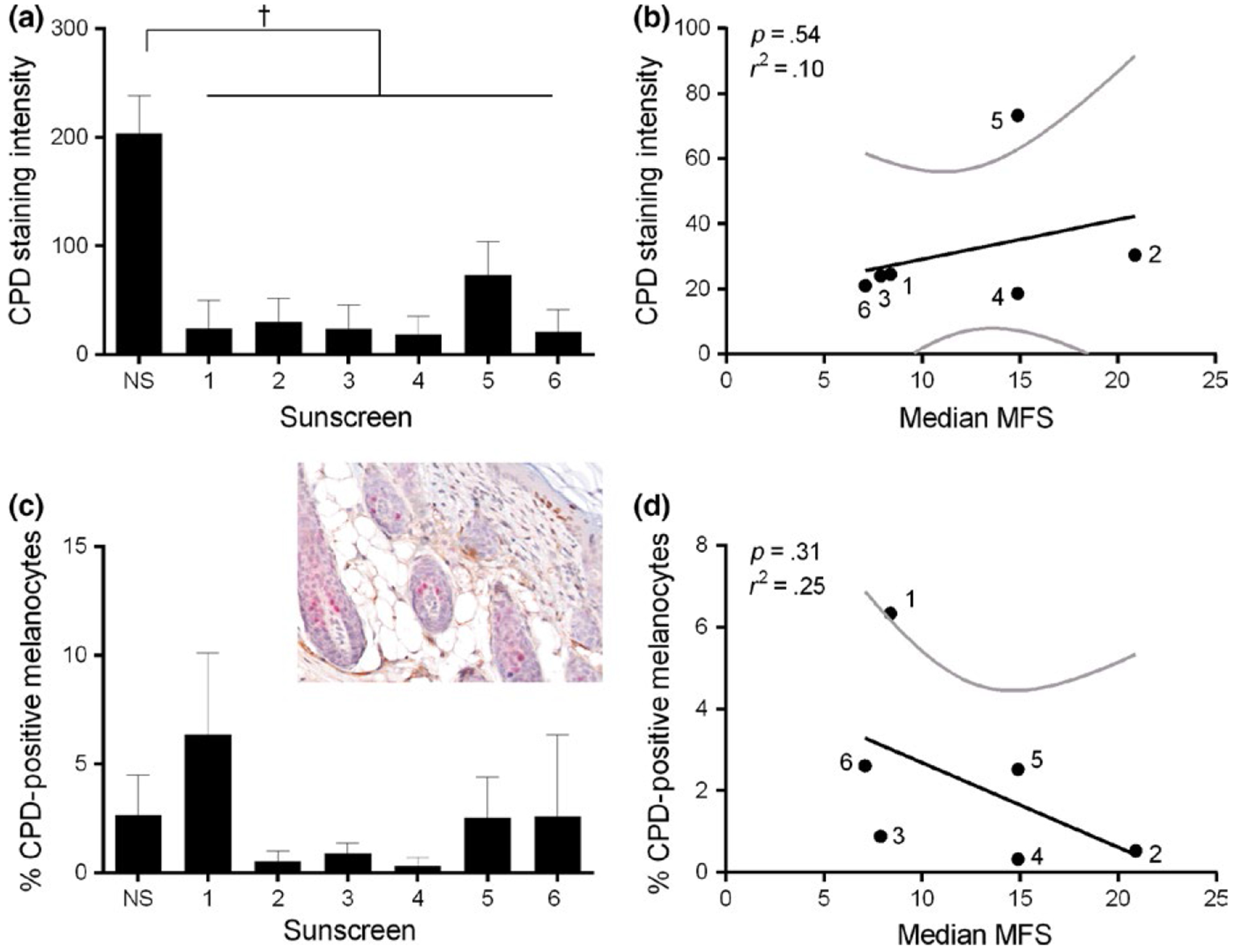
Differences in DNA damage protection do not correlate with sunscreen efficacy. (a) Quantification of total cyclobutane pyrimidine dimer (CPD) staining intensity in sunscreen-treated *TpN^61R^* skins six hours post-UV. CPD staining was quantified using inForm 2.0.2 software H-score analysis, which calculates the relative intensity of nuclear staining across all replicates, assigning each image a score between 0 (no staining) to 300 (all cells intensely stained). Data shown represent the mean ± standard deviation of four quantified skin images per mouse with three to four mice per treatment group. ^†^*p* ≤ .0001 as determined by comparisons of the “No sunscreen” group to other groups using two-sided, unpaired t tests with Welch’s correction. (b) Linear regression of average CPD staining intensity in sunscreen-treated *TpN^61R^* skins six hours post-UV versus median MFS (see Table 1). Numbers correspond to individual sunscreen products. 95% confidence intervals are shown in gray. (c) Quantification of the percent of CPD-positive melanocytes in skin dually stained for MITF (melanocyte marker) and CPD six hours post-UV administration. Positivity data are expressed as the mean percentage ± standard deviation of CPD-positive melanocytes detected within ten fields of view from three to five biological replicates. Representative image of dual CPD (brown) and MITF (red) staining appears in the upper right corner. No significant differences were detected between the “No sunscreen” group and any other group using two-sided, unpaired t tests with Welch’s correction. (d) Linear regression comparing the percentage of CPD-positive melanocytes in sunscreen-treated *TpN^61R^* skins six hours post-UV to median MFS (see Table 1). Numbers correspond to individual sunscreen products. 95% confidence intervals are shown in gray [Colour figure can be viewed at wileyonlinelibrary.com]

The extension of MFS was not equivalent among sunscreens. Delays in tumor onset were least dramatic for sunscreens one, three, and six, and most dramatic for sunscreen two (Table 1). Sunscreen-dependent increases in MFS did not correlate with marketed U.S. or calculated in silico SPF values (Figure 3a, Table 1). The ability of chemical sunscreens to prevent melanoma was also unrelated to absorbance potential within the spectral range of the UVB-centered light source (Figure 3b,c). While our studies were not designed or powered to detect changes in sunscreen efficacy related to specific active ingredients, we looked for potential correlations between product composition and MFS. When corrected for application amount (see Methods), none of the active ingredients studied correlated with the ability of a sunscreen to delay melanoma onset; however, sunscreens with higher homosalate or avobenzone levels showed a trend toward better performance (Figure S2; *p* = .067 and .074, respectively). While all sunscreens blocked peak cyclobutane pyrimidine dimer (CPD) formation in dorsal skin samples procured six hours after UV exposure, differences in residual DNA damage throughout the skin or specifically in melanocytes did not correlate with the melanoma preventative efficacy of each product (Figure 4, Figure S3, and Table 1). Therefore, DNA damage-independent effects influence the ability of sunscreens to block UVB-dependent melanomagenesis.

### 2.5 | Sunscreen coverage determines melanoma preventative efficacy

**FIGURE 5:**
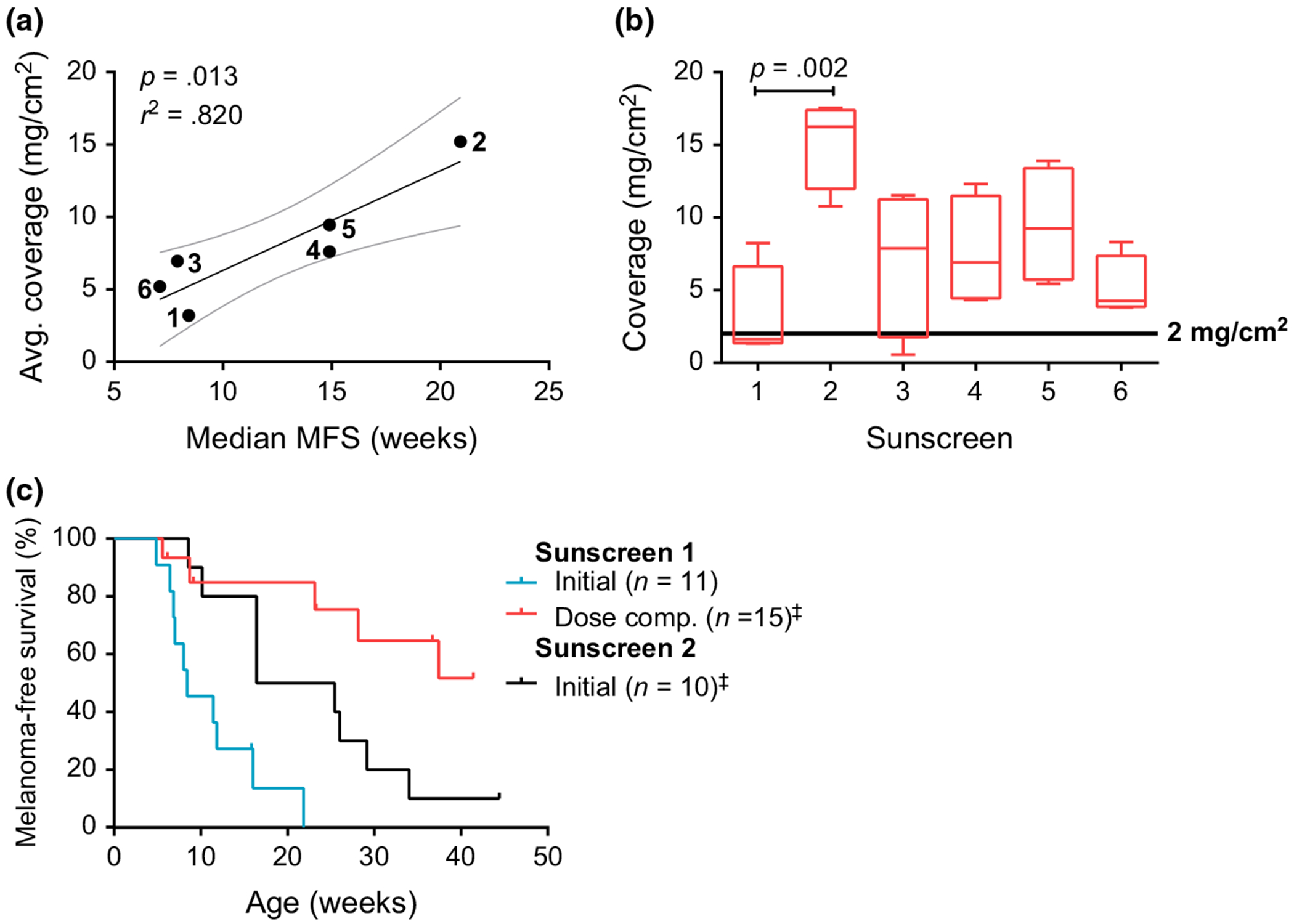
Aerosol spray coverage correlates with melanoma preventative efficacy. (a) Linear regression of experimental sunscreen coverage versus median melanoma-free survival (see Table 1). (b) Experimental coverage of sunscreens relative to international testing standards (2 mg/cm^2^). Data shown as box and whiskers plot with whiskers representing 5 and 95 percentiles of four technical replicates. (c) Melanoma-free survival of UV-exposed *TpN^61R^* mice treated with Sunscreen 1 (initial), four times the amount of Sunscreen 1 (dose compensated), or Sunscreen 2 (initial). ^‡^*p* ≤ .01 [Colour figure can be viewed at wileyonlinelibrary.com]

Although consumers cannot alter the chemical composition of sunscreens, they can control the type, frequency, and amount of product applied. To test whether sunscreen coverage (i.e., applied amount per cm^2^) improved performance in our model, we measured the output of each aerosol sunscreen. We found that spray output correlated directly with median MFS (Figure 5a, *p* = .012), even though sunscreen coverage under the test parameters met or exceeded current international recommendations (i.e., 2 mg/cm^2^; Figure 5b). Further examination of aerosol sunscreen output revealed dramatic lot-to-lot variations in coverage (Figure S4a). Moreover, in many cases product delivery was significantly lower when tested early during use versus mid-way through the bottle (Figure S4b). Of note, our in vivo assays were conducted with products directly off the shelf and used a minimal amount of product (no more than eight short-pass sprays); therefore, early readings were used to estimate the applied volume of sunscreen per mouse.

To establish how sunscreen coverage impacts MFS, we focused on the differential melanoma preventative efficacy of sunscreens one and two (median MFS of 8.4 and 20.9 weeks, respectively; *p* = .005, Table 1). When applied at a coverage equivalent to that of sunscreen two, the performance of sunscreen one improved significantly (Figure 5c; *p* = .003 compared to initial sunscreen 1; *p* = .11 compared to sunscreen 2). The MFS, OS, and average tumor burden of *TpN^61R^* mice treated with dose-compensated sunscreen one versus sunscreen two were similar, demonstrating that coverage—not composition—is the major determinant of sunscreen efficacy in our model (Figure 5c; Table 1).

## 3 | DISCUSSION

Sunscreen manufacturing is a growing, 399 million dollar industry with some of the most popular products being aerosol sprays of SPF 15 or higher (Carter, 2015; Osterwalder, Sohn, & Herzog, 2014). While current clinical recommendations may be driving the demand for sunscreens with an SPF greater than 15 (e.g., the American Academy of Dermatology recommends SPF 30+) (American Academy of Dermatology), there are no data to support the claim that any specific sunscreen formulation provides superior protection from melanoma. Moreover, limited understanding of how sunlight mechanistically promotes melanoma onset reduces our ability to identify key molecular targets. The expectation that case–control studies might be conducted to assess the melanoma preventative potential of individual sunscreen formulations is unrealistic. To recapitulate known epidemiological links between childhood sunburn, intermittent sun exposure, and lifetime melanoma risk would require decades of follow-up in large cohorts. Even if such a study was financially and logistically feasible, assuring long-term compliance, sufficient sunscreen application, and consistent product selection is unrealistic. By capitalizing on the exceptional UV sensitivity of our clinically relevant *TpN^61R^* model, we have developed a rapid in vivo testing method in which the melanoma preventative efficacy of distinct sunscreen formulations can be directly compared.

Several distinguishing features of the *TpN^61R^*-UV model make it physiologically relevant for studies of sunscreen efficacy. First, the presence of *NRAS* in human spontaneous and congenital nevi indicates that these early genetic events are insufficient to initiate tumorigenesis (Bauer et al., 2007; Ross et al., 2011; Shain et al., 2015). Given the well-established epidemiological link between intermittent sun exposure and melanoma risk (Gandini et al., 2005; Whiteman et al., 2001), the fact that melanoma-associated *NRAS* mutations are UV-independent (Cancer Genome Atlas Network, 2015) suggests that UV promotes secondary events essential for tumor initiation. Indeed, marked cooperativity between NRas^61R^ and UV in driving *TpN^61R^* melanomas supports this argument. Finally, it is important to note that NRas^61R^ is expressed from the endogenous gene locus in *TpN^61R^* mice, as transgenic overexpression can lead to protein mislocalization and aberrant downstream signal transduction (Omerovic & Prior, 2009; Pylayeva-Gupta et al., 2011). Thus, even at the molecular level, *TpN^61R^* mice closely replicate NRAS-mutant human melanocytes.

Here, we suggest that the prevalence of codon 61 alterations in NRAS-mutant human melanoma could be explained by the unique ability of these alleles to cooperate with a single dose of UV in initiating tumorigenesis. To this end, we find that although a single dose of UV accelerates melanoma onset by 80% in the *TN^61R^* model, this same exposure fails to impact tumor formation in animals harboring an *NRas^12D^* allele (Figure 2). However, one report suggests that NRas^12D^ can function in concert with long-term, chronic UV dosing to facilitate melanoma formation (Viros et al., 2016). Unfortunately, this study did not include an NRas^61R^-mutant model and therefore, further work is needed to determine whether NRas codon 12 and 61 alterations equally promote melanoma formation in the context of chronic UV administration. Such a study would be important, as epidemiological data link NRAS-mutant melanomas to older cohorts with presumably higher levels of cumulative sun damage (Edlundh-Rose et al., 2006; Ekedahl et al., 2013; Hacker et al., 2013; Thomas et al., 2015). Nevertheless, sequencing by The Cancer Genome Atlas (TCGA) finds UV signature mutations to be equally prevalent among BRAF-, NF1-, and NRAS-mutant melanomas, which exhibit distinct ages of onset (Cancer Genome Atlas Network, 2015). Furthermore, patients with chronic sun damage are not necessarily at higher risk for developing NRAS-mutant melanomas over other genetic subtypes (Devitt et al., 2011; Sakaizawa et al., 2015; Yaman, Akalin, & Kandiloglu, 2015), and lentigo maligna melanomas, which are most strongly associated with UV damage, are typically *NRAS* wild type (Devitt et al., 2011; Thomas et al., 2015; Yaman et al., 2015). For these reasons, we surmise that chronic UV damage is not requisite to the formation of NRAS-mutant melanomas and suggest that the later onset of these tumors in humans may reflect genotype-specific differences in the probability of any one UV-event to trigger transformation (see below). However, studies to determine whether a single, neonatal UV exposure can more effectively accelerate BRaf-mutant melanoma initiation are just beginning in the lab. Finally, we show that p16^INK4a^ loss, a common genetic event in melanoma, accelerates tumor growth and facilitates initiation, but is not the major cooperating factor in the promotion of melanoma formation by UV (Figure 2a–c).

The dose of UV required to accelerate melanomagenesis in the *TpN^61R^* model is biologically relevant. For example, the minimal erythemic dose for humans with skin type II on the Fitzpatrick scale (i.e., skin that burns easily and rarely tans) is 210 J/m^2^ EEE (Mckinlay & Diffey, 1987). The median MFS of *TpN^61R^* mice was reduced following exposure to single dose of 420 J/m^2^ EEE UVB (2.3 kJ/m^2^ UV, non-weighted; Figure 1b). Moreover, at no experimental dose did we see evidence of erythema, edema, or blistering (data not shown). Together, these data show that even a single, non-erythematous dose of UV radiation can facilitate the transformation of genetically primed nevi—a compelling finding to support daily sunscreen use regardless of anticipated outdoor activity. While the observation that a single UV exposure is able to trigger melanoma onset seems unfathomable, it is important to note that nearly all melanocytes in the *TpN^61R^* and *TN^61R^* models are genetically predisposed to tumor formation, yet these mice never develop more than nine tumors (Figure 1d). This finding shows that there is a degree of stochasticity in UV-triggered melanoma initiation. As the frequency of *NRAS* mutations in human melanocytes is considerably less than in our murine models, we propose that the probability of a single sun exposure leading to transformation remains low. Nevertheless, our data provide evidence that every sun exposure is capable of driving NRAS-mutant melanocyte transformation.

Having experimentally established the incredible sensitivity of *NRas*-mutant melanoma precursor lesions to UV-induced transformation, the need to assess what constitutes an effective sunscreen became evident. Currently, international sunscreen testing focuses on the ability of a product to block UV transmittance (e.g., broad spectrum), resist water emersion (e.g., water resistance), and prevent UVB-mediated erythema (i.e., sunburn (SPF)) (Food and Drug Administration 2011; International Organization for Standardization 2010). No premarket testing determines the ability of a sunscreen to prevent melanoma, the deadliest form of skin cancer.

Despite concerns about their flammability and dangers as inhalants, aerosols are some of the most popular sunscreens among the general public because they are easy to apply. While our studies show that a wide variety of aerosol sunscreens effectively prevent the acceleration of melanoma formation caused by UV exposure, our findings also raise concerns regarding the variable output of these products during use and from lot to lot (Figure S4). This observation is especially concerning because of the direct correlation between melanoma preventative activity and sunscreen coverage in our model (Table 1, Figure 5).

Although our studies were not powered to test for differences in sunscreen composition, levels of homosalate and avobenzone trended toward a positive correlation with median MFS (Figure S2, *p* = .067, *p* = .074). One caveat of these analyses is that sunscreen performance is measured over a compressed period of time and may not accurately capture the long-term stability of active ingredients. Future studies employing a light source with reduced intensity may mitigate this issue, providing a more physiological relevant testing scheme. Nevertheless, as avobenzone is a UVA blocking agent and our filtered light source maintains some UVA, we were intrigued by the putative correlation between avobenzone levels and prolonged MFS (Figure S2). To investigate this more thoroughly, we treated *TpN^61R^* mice with an aerosolized SPF10 sunscreen containing no UVA blocking components (homosalate 5.0%, octinoxate 7.5%, octisalate 5.0%, oxybenzone 3.0%). Both melanoma-free and overall survival were significantly extended by this product (MFS > 17.1 weeks, OS > 17.1 weeks, *p* = .0019, *n* = 6), suggesting that UVA is not the major driver of tumorigenesis in our current system (data not shown). Given that inactive sunscreen components are implicated in both photocarcinogenesis (e.g., retinyl palmitate/vitamin A) and protection (e.g., tocopherol/vitamin E) (Burnett & Wang, 2011), we attempted to establish whether other “inactive” sunscreen components contributed to a products’ ability to prevent melanoma. Unfortunately, the complexity and proprietary nature of our selected sunscreen products prohibited us from making any claims regarding the role of excipients, fragrances, or film-forming agents in melanoma prevention.

While mineral sunscreens are rarely manufactured as aerosols due to concerns about particle inhalation, we were able to identify one product to test in our model. Sunscreen 6 is a non-nanoparticle, allnatural product that lists only one active ingredient—12% zinc oxide. While this product was among the poorest performing sunscreens in our study, it is important to note that this sunscreen exhibited poor coverage and might show increased efficacy with further application. Notably, an aerosolized 10% zinc oxide rash cream was effective in delaying melanoma onset in our model (median MFS = 8.7 weeks, *n* = 12, *p* = .002; coverage = 11.8 ± 2.7 mg/cm^2^, data not shown). Together, these data support the idea that a variety of sunscreen formulations, when applied liberally, protect against melanoma formation. Whether the amount of sunscreen required to fully protect mice is practical and reproducible using current product delivery methods is unclear. Our findings encourage sunscreen coverage exceeding current recommendations (i.e., 2 mg/cm^2^), yet most consumers fail to apply even this amount (Diffey, 2001). Knowing this, some physicians recommend the use of sunscreens with higher SPF ratings to compensate for poor coverage; however, it is important to mention that SPF is not a linear measurement and therefore applying 50% less than the recommended amount of an SPF 50 sunscreen does not necessarily result in the same protection as an appropriately applied SPF 30 product.

The *TpN^61R^*-UV model opens up new avenues for the exploration of critical questions in melanoma prevention. Here, we find that the protective effects of some sunscreens extend beyond DNA damage prevention. Sunscreens providing variable protection from UV-accelerated melanomagenesis were equally capable of preventing DNA damage (Figure 4, Figure S3, and Table 1). Greater understanding of these non-genomic mechanisms could greatly improve sunscreen performance. The *TpN^61R^*-UV model could also be used to identify the UV action spectrum for melanoma by defining which wavelengths of UV light are the most melanomagenic. Such information would aid in sunscreen design, allowing for the development of simpler product formulations targeting only the most critical, specific UV wavelengths. Beyond efficacy, sunscreen toxicity, biodistribution, allergenic potential, and endocrine action could be simultaneously evaluated in the *TpN^61R^*-UV model.

It remains to be determined whether other murine melanoma models could be included in such in vivo testing. Chronic UV administration is known to accelerate melanomagenesis in adult, *Braf*-mutant mice (Viros et al., 2014), but the effects of neonatal exposure have yet to be reported. In adult *Braf*-mutant mice, the accelerated onset of melanoma is partially mitigated by at least one sunscreen formulation (Viros et al., 2014). Likewise, transgenic mice expressing melanocytic HGF have been used to test sunscreen efficacy in melanoma prevention (Klug et al., 2010). Of note, these past models do not comprehensively recapitulate the endogenous genetics, progression, and sensitivity to intermittent UV documented in human melanoma like the *TpN^61R^*-UV system. Furthermore, our work demonstrates that this model has unique utility in that rapid, in vivo screens of distinct sunscreen formulations can be readily conducted to identify effective melanoma preventatives.

This work supports several clinical paradigms. First, that daily sunscreen use may be necessary in patients with genetically primed melanocytic lesions. Second, that sufficient sunscreen application is the main determinant of melanoma preventative efficacy when product SPF ratings meet or exceed 30. Knowing that most consumers apply less than the recommended amount of sunscreen (Diffey, 2001; Reich, Harupa, Bury, Chrzaszcz, & Starczewska, 2009; Schalka, Dos Reis, & Cuce, 2009), patient education regarding sunscreen application is essential. With so many modes of application, this may be a challenge—especially when aerosol sunscreen product output is clearly inconsistent. Finally, we hope that this work will put to rest the controversy surrounding sunscreen efficacy in melanoma prevention by showing that a wide variety of commercial sunscreens protect against UV-induced melanoma formation in a physiologically relevant, in vivo model.

## 4 | METHODS

### 4.1 | Study subjects

All animal research protocols were reviewed and approved by Ohio State’s Institutional Animal Care and Use Committee (Protocol #2012A00000134). The *TpN^61R^* and *TpN^12D^* models employ a tamoxifen-inducible transgenic allele (*Tyr::CreER(T2)*) to drive melanocyte-specific CRE recombinase activity (Bosenberg et al., 2006; Burd et al., 2014). In these models, the endogenous *NRas* and *CDKN2a* loci are modified such that CRE activation leads to the production of mutant NRas^61R^ or NRas^12D^ and loss of p16^INK4a^ expression (Figure 1a). The *Tp* and *TN^61R^* models are derivatives generated by selectively breeding *TpN^61R^* mice. These models lack the *LSL-NRas^61R^* or *p16^L^* allele, respectively. All mice were backcrossed at least seven generations to C57Bl/6J, a highly pigmented mouse strain.

### 4.2 | Exposure to ultraviolet radiation

Inducible knock-in and knockout alleles were activated with 20 mM 4-hydroxytamoxifen on post-natal (p.n.) days one and two as described (Burd et al., 2014). Subjects from the each litter were randomly assigned to be exposed dorsally to UV doses ranging from 0 to 9 kJ/m^2^ on p.n. day three using a fixed position, 16W, 312 nm UV light source (Spectronics #EB-280C). At this age, mice are unfurred and therefore use of a depilatory agent is unnecessary. The spectral distribution of our experimental light source is shown in Figure 3b and is predominantly weighted toward the UVB spectrum (280–315 nm) with some UVA (315–400 nm) and almost no UVC (100–280 nm) emission. The length of UV treatment was determined using a UVX digital radiometer and UVX-31 (302 nm) sensor and ranged between four and 154 seconds, depending on the administered dose. Radiation intensity at the exposure site was 60 W/m^2^; therefore, in our system, the McKinlay–Diffey erythemal effective energy (EEE) (Mckinlay & Diffey, 1987) of a 4.5 kJ/m^2^ exposure is 750 J/m^2^ or 75 mJ/cm^2^. This dose is equivalent to 3.9 human minimal erythema doses (MEDs) in an individual with phototype II skin (i.e., someone who tans minimally, but usually burns with red/blond hair and blue/green/hazel eyes) or to approximately 40 min of sunbathing when the UV index is very high (i.e., peak summer sun).

### 4.3 | Immunohistochemistry

To detect cyclobutane pyrimidine dimer formation in the skin, rehydrated paraffin-embedded tissue sections were incubated in 0.3% H_2_O_2_ in 100% methanol for 10 min at room temperature, subjected to heat-mediated antigen retrieval in citrate buffer (DAKO #S1699), and then treated with 1 N HCl for 30 min at room temperature. To prevent background staining, primary antibody (i.e., antithymine dimer antibody; Kamiya Biomedical Co., MC062 at 1:10) was applied using M.O.M. reagents (Vector Laboratories). Following incubation, staining was detected with ABC reagent (Vector Laboratories) and diaminobenzadine (DAB) solution (DAKO). Slides were counterstained with hematoxylin, dehydrated, and mounted. To stain for cyclobutane pyrimidine dimer formation in melanocytes, the DAB-based protocol above was followed by melanin bleaching in 10% H_2_O_2_ in PBS at 60°C for 90 min and then incubation in 2.5% horse serum at room temperature for 20 min. Next, tissue sections were incubated with anti-MITF primary antibody (Abcam, ab122982 at 1:1,000) followed by detection with ImPRESS-AP reagent (Vector Laboratories) and Fast Red (ThermoFisher Scientific). Slides were counterstained, dehydrated and mounted before analysis with inForm2.0.2 software (PerkinElmer). p53 was detected by standard IHC methods, including heat-mediated antigen retrieval in citrate buffer (DAKO S1699), protein blocking (DAKO X0909), incubation with primary antibody (Leica NCL-L-p53-CM5p at 1:500), and subsequent detection using biotinylated secondary antibody, ABC reagent and DAB solution. In all cases, staining intensity (H-score) was quantified using inForm2.0.2 software (PerkinElmer). First, hematoxylin counterstaining was used to define nuclei. Next, the relative intensity of nuclear DAB staining (i.e., H-score) was determined by the software and ranged between 0 (no staining) and 300 (all cells intensely stained). For melanocyte CPD positivity, Fast Red (MITF)-positive nuclei were first detected and then scored for the absence or presence of CPD staining (DAB) using inForm software.

### 4.4 | Real-time PCR analysis

Levels of *p16^INK4a^* mRNA were quantified as described (Burd et al., 2013).

### 4.5 | Sunscreen testing and coverage measurements

Aerosol sunscreens were purchased from commercial vendors and used prior to expiration. The sunscreen product used for each litter was predetermined prior to birth. Littermates were randomly assigned to be treated with sunscreen or no sunscreen. Before UV exposure, mice receiving sunscreen were placed, dorsal side up, together in a sterile, 60-mm^2^ petri dish. Holding the bottle approximately four inches from the animals (to replicate consumer use), researchers applied a consistent pass of sunscreen spraying once from left to right across the dish and once from top to bottom. [Note that initiation of the spray began prior to contact with the petri dish.] Sunscreen coverage was determined by replicating the application procedure above in the absence of mice. The weight of sunscreen captured in the empty 60-mm^2^ petri dish was determined and used to calculate product output per area (mg/cm^2^). Measurements were repeated on several occasions with different laboratory staff members performing the application procedure.

### 4.6 | Outcome monitoring

Subjects were randomly numbered following treatment and blindly monitored biweekly for tumor formation. Established melanomas were measured daily by caliper and tumor size (width × length (mm)) recorded until protocol exclusion criteria were met. Upon sacrifice, numbered mice were de-identified.

### 4.7 | Statistical analysis

Kaplan–Meier survival analyses and Wilcoxon tests were used to compare melanoma-free and overall survival between study groups. Sample sizes for survival analyses were determined to detect a one standard deviation change in median survival between groups (α = .05, 80% power) based upon pilot data comparing 4.5 kJ/m^2^ UV to no UV treatment in *TpN^61R^* mice. Differences in tumor burden, tumor onset, tumor growth rate, experimental spray coverage, and CPD and p53 intensity were determined using unpaired, two-sided t tests with Welch’s correction. CPD and p53 sample sizes were powered to detect a twofold difference in study groups based upon prior skin and tumor staining results in 4.5 kJ/m^2^ UV to no UV treatment in *TpN^61R^* mice (α = .05, 80% power, CV = 30%, two-sided). Slope comparisons were assessed by ANCOVA, and correlations between variables were determined using linear regression.

## Supplementary Material

legends

SOP figures

Additional Supporting Information may be found online in the supporting information tab for this article.

## Figures and Tables

**TABLE 1:** Evaluation of commercial sunscreen efficacy

	No Sunscreen	Sunscreen 1	Sunscreen 2	Sunscreen 3	Sunscreen 4	Sunscreen 5	Sunscreen 6
Sunscreen properties	*n* = 22	*n* = 11	*n* = 10	*n* = 9	*n* = 11	*n* = 13	*n* = 17
**Active Ingredient (%)**							

Avobenzone	–	3	3	3	3	2	–

Homosalate	–	10	10	8.8	8	7	–

Octisalate	–	4.5	5	4.9	5	–	–

Oxybenzone	–	2	4	–	5	–	–

Octocrylene	–	9	2	5.9	4	5	–

Zinc Oxide	–	-	–	–	–	–	12

**Bioactive excipients**							

Tocopherol (Vitamin E)	–	YES	YES	YES	YES	YES	–

Ascorbyl palmitate (Vitamin C)	–	YES	–	–	–	YES	–

**SPF rating**							

U.S. Marketed SPF	–	50	30	30	30	30	30

BASF in silico	–	26.9	20.1	19.2	23.6	12.9	ND

**Outcome data**							

Melanoma-free survival (weeks)	5.3	**8.4** ^‡^	**20.9** ^Δ^	**7.9** ^‡^	**14.9** ^‡^	**14.9** ^Δ^	**7.1** *

Overall survival (weeks)	9	**14.4** ^‡^	**26.9** ^Δ^	**15.7** *	**16.6** ^Δ^	**24.0** ^Δ^	12.7

**Tumor statistics**							

Tumor burden (avg. #/mouse ± *SD*)	4.4 ± 4.7	**1.4 ± 0.52** *	**1.8 ± 0.97** *	2.1 ± 1.6	**1.2 ± 0.41** *	**1.7 ± 1.0** *	2.2 ± 2.2

Tumor growth rate (% Δ size ± *SD*)	211.4 ± 269.0	178.4 ± 98.1	96.4 ± 69.5	134.5 ± 108.6	139.6 ± 66.44	140.1 ± 140.0	187.4 ± 234.6

**CPD staining**							

Total skin intensity at 6 hr (±*SD*)	203.5 ± 34.8	**24.5 ± 25.3** ^†^	**30.4 ± 21.4** ^†^	**24.0 ± 21.8** ^†^	**18.6 ± 16.7** ^†^	**73.3 ± 30.6** ^†^	**21.1 ± 20.6** ^†^

Melanocyte positivity at 6 hr (±*SD*)	2.6 ± 1.9	6.3 ± 3.7	0.53 ± 0.48	0.88 ± 0.46	0.32 ± 0.36	2.5 ± 1.9	2.6 ± 1.9

**Sunscreen output data**							

Experimental coverage (mg/cm^2^ ± *SD*)	–	3.2 ± 3.4	15.2 ± 3.1	7.0 ± 5.0	7.6 ± 3.8	9.5 ± 4.1	5.2 ± 2.1

***Sunscreen properties***, Active ingredient concentrations and the presence of bio-active excipients are indicated for each sunscreen. BASF in silico ratings are based upon active ingredient concentrations and were determined using the online BASF sunscreen simulator (https://www.sunscreensimulator.basf.com/) ***Outcome data***, The median time to an event (melanoma onset or mortality) is indicated in weeks. *p*-values were calculated versus “No Sunscreen” using Wilcoxon tests. ***Tumor statistics***, Tumor burden indicates the average number of tumors per mouse at the time of euthanasia. Tumor growth was determined by calculating the slope of the linear regression of tumor size over time for each melanoma. Values represent the average slope for tumors within the indicated treatment cohort. *p*-values were calculated versus “No Sunscreen” using unpaired, two-sided t tests with Welch’s correction. ***CPD staining***, Average cyclobutane pyrmidine dimer (CPD) staining intensity (i.e., H-score) was determined in total skin nuclei from at least 12 images using in-Form 2.0 software. Melanocyte positivity was calculated using inForm software to score the percentage of MITF-positive cells (melanocytes) staining positive for CPD at six hours post-UV treatment in ten images per mouse with three to five mice per treatment. p-values were calculated versus “No Sunscreen” using unpaired, two-sided t tests with Welch’s correction. ***Sunscreen output data***, The average experimental spray coverage for each product was determined after treatment of the study animals as indicated in the Methods. Bolded values indicate statistical significance versus the no sunscreen control.

* *p* ≤ .05,

‡ *p* ≤ .01,

Δ = *p* ≤ .001,

† *p* ≤ .0001;

ND = Not determined.

## Significance

Our data provide one rationale for the high frequency of NRas codon 61 mutations in melanoma, showing that other tumor-associated alleles (i.e., NRas^12D^ and p16^INK4a^ loss) fail to initiate melanoma formation in cooperation with a single UV exposure. It is well established that consumers apply less than the recommended amount of sunscreen, and our data provide experimental evidence that sunscreen application has a greater impact on the efficacy of SPF30 products than product formulation. Furthermore, we establish utility for the *TpN^61R^* model in rapidly assessing next-generation melanoma preventatives targeting both the genomic and non-genomic effects of UV.

## References

[R1] American Academy of Dermatology. What sunscreen should I use? In Sunscreen FAQs. Retrieved from https://www.aad.org/public/spot-skin-cancer/learn-about-skin-cancer/prevent/how-to-select-a-sunscreen

[R2] Bauer J, Curtin JA, Pinkel D, & Bastian BC (2007). Congenital melanocytic nevi frequently harbor NRAS mutations but no BRAF mutations. The Journal of Investigative Dermatology, 127, 179–182. 16888631 10.1038/sj.jid.5700490

[R3] Bosenberg M, Muthusamy V, Curley DP, Wang Z, Hobbs C, Nelson B, … Chin L (2006). Characterization of melanocyte-specific inducible Cre recombinase transgenic mice. Genesis, 44, 262–267. 16676322 10.1002/dvg.20205

[R4] Burd CE, Liu W, Huynh MV, Waqas MA, Gillahan JE, Clark KS, … Sharpless NE (2014). Mutation-specific RAS oncogenicity explains NRAS codon 61 selection in melanoma. Cancer Discovery, 4, 1418–1429. 25252692 10.1158/2159-8290.CD-14-0729 PMC4258185

[R5] Burd CE, Sorrentino JA, Clark KS, Darr DB, Krishnamurthy J, Deal AM, … Sharpless NE (2013). Monitoring tumorigenesis and senescence in vivo with a p16(INK4a)-luciferase model. Cell, 152, 340–351. 23332765 10.1016/j.cell.2012.12.010 PMC3718011

[R6] Burnett ME, & Wang SQ (2011). Current sunscreen controversies: A critical review. Photodermatology, Photoimmunology & Photomedicine, 27, 58–67. 10.1111/j.1600-0781.2011.00557.x 21392107

[R7] Cancer Genome Atlas Network (2015). Genomic classification of cutaneous melanoma. Cell, 161, 1681–1696. 26091043 10.1016/j.cell.2015.05.044 PMC4580370

[R8] Carter B (2015). IBISWorld industry report OD4244: Sunscreen manufacturing in the US. In US Specialized Industry Reports: IBISWorld. Los Angles, CA: IBIS World, Inc.

[R9] Devitt B, Liu W, Salemi R, Wolfe R, Kelly J, Tzen CY, … Mcarthur G (2011). Clinical outcome and pathological features associated with NRAS mutation in cutaneous melanoma. Pigment Cell & Melanoma Research, 24, 666–672. 21615881 10.1111/j.1755-148X.2011.00873.x

[R10] Diffey B (2001). Sunscreen isn’t enough. Journal of Photochemistry and Photobiology. B, Biology, 64, 105–108. 11744396 10.1016/s1011-1344(01)00195-6

[R11] Edlundh-Rose E, Egyhazi S, Omholt K, Mansson-Brahme E, Platz A, Hansson J, & Lundeberg J (2006). NRAS and BRAF mutations in melanoma tumours in relation to clinical characteristics: A study based on mutation screening by pyrosequencing. Melanoma Research, 16, 471–478. 17119447 10.1097/01.cmr.0000232300.22032.86

[R12] Ekedahl H, Cirenajwis H, Harbst K, Carneiro A, Nielsen K, Olsson H, … Jonsson G (2013). The clinical significance of BRAF and NRAS mutations in a clinic-based metastatic melanoma cohort. The British Journal of Dermatology, 169, 1049–1055. 23855428 10.1111/bjd.12504

[R13] Food and Drug Administration, H. H. S. (2011). Labeling and effectiveness testing; sunscreen drug products for over-the-counter human use. Final rule. Federal register, 76, 35620–35665. 21682059

[R14] Forbes SA, Beare D, Gunasekaran P, Leung K, Bindal N, Boutselakis H, … Campbell PJ (2015). COSMIC: Exploring the world’s knowledge of somatic mutations in human cancer. Nucleic Acids Research, 43, D805–D811. 25355519 10.1093/nar/gku1075 PMC4383913

[R15] Gandini S, Sera F, Cattaruzza MS, Pasquini P, Picconi O, Boyle P, & Melchi CF (2005). Meta-analysis of risk factors for cutaneous melanoma: II. Sun exposure. European Journal of Cancer, 41, 45–60. 15617990 10.1016/j.ejca.2004.10.016

[R16] Green AC, Williams GM, Logan V, & Strutton GM (2011). Reduced melanoma after regular sunscreen use: Randomized trial follow-up. Journal of Clinical Oncology: Official Journal of the American Society of Clinical Oncology, 29, 257–263. 21135266 10.1200/JCO.2010.28.7078

[R17] Green A, Williams G, Neale R, Hart V, Leslie D, Parsons P, … Russell A (1999). Daily sunscreen application and betacarotene supplementation in prevention of basal-cell and squamous-cell carcinomas of the skin: A randomised controlled trial. Lancet, 354, 723–729. 10475183 10.1016/S0140-6736(98)12168-2

[R18] Hacker E, Nagore E, Cerroni L, Woods SL, Hayward NK, Chapman B, … Whiteman DC (2013). NRAS and BRAF mutations in cutaneous melanoma and the association with MC1R genotype: Findings from Spanish and Austrian populations. The Journal of Investigative Dermatology, 133, 1027–1033. 23096702 10.1038/jid.2012.385

[R19] Hodis E, Watson IR, Kryukov GV, Arold ST, Imielinski M, Theurillat JP, … Chin L (2012). A landscape of driver mutations in melanoma. Cell, 150, 251–263. 22817889 10.1016/j.cell.2012.06.024 PMC3600117

[R20] Iannacone MR, Hughes MC, & Green AC (2014). Effects of sunscreen on skin cancer and photoaging. Photodermatology, Photoimmunology & Photomedicine, 30, 55–61. 10.1111/phpp.12109 24417448

[R21] International Organization for Standardization (2010). Cosmetics—Sun protection test methods—In vivo determination of the sun protection factor (SPF).

[R22] Klug HL, Tooze JA, Graff-Cherry C, Anver MR, Noonan FP, Fears TR, … Merlino G (2010). Sunscreen prevention of melanoma in man and mouse. Pigment Cell & Melanoma Research, 23, 835–837. 20726949 10.1111/j.1755-148X.2010.00756.x PMC2995311

[R23] Maslin DL (2014). Do suncreens protect us? International Journal of Dermatology, 53, 1319–1323. 25208462 10.1111/ijd.12606

[R24] Mckinlay AF, Diffey BL (1987). A reference action spectrum for ultraviolet induced erythema in human skin. CIE Journal, 6, 17–22.

[R25] Mukhopadhyay P, Ferguson B, Muller HK, Handoko HY, & Walker GJ (2016). Murine melanomas accelerated by a single UVR exposure carry photoproduct footprints but lack UV signature C>T mutations in critical genes. Oncogene, 35, 3342–3350. 26477315 10.1038/onc.2015.386

[R26] Omerovic J, & Prior IA (2009). Compartmentalized signalling: Ras proteins and signalling nanoclusters. The FEBS Journal, 276, 1817–1825. 19243428 10.1111/j.1742-4658.2009.06928.x

[R27] Osterwalder U, Sohn M, & Herzog B (2014). Global state of sunscreens. Photodermatology, Photoimmunology & Photomedicine, 30, 62–80. 10.1111/phpp.12112 24734281

[R28] Pollock PM, Harper UL, Hansen KS, Yudt LM, Stark M, Robbins CM, … Meltzer PS (2003). High frequency of BRAF mutations in nevi. Nature Genetics, 33, 19–20. 12447372 10.1038/ng1054

[R29] Poynter JN, Elder JT, Fullen DR, Nair RP, Soengas MS, Johnson TM, … Gruber SB (2006). BRAF and NRAS mutations in melanoma and melanocytic nevi. Melanoma Research, 16, 267–273. 16845322 10.1097/01.cmr.0000222600.73179.f3

[R30] Pylayeva-Gupta Y, Grabocka E, & Bar-Sagi D (2011). RAS oncogenes: Weaving a tumorigenic web. Nature Reviews. Cancer, 11, 761–774. 21993244 10.1038/nrc3106 PMC3632399

[R31] Reich A, Harupa M, Bury M, Chrzaszcz J, & Starczewska A (2009). Application of sunscreen preparations: A need to change the regulations. Photodermatology, Photoimmunology & Photomedicine, 25, 242–244. 10.1111/j.1600-0781.2009.00450.x 19747242

[R32] Ross AL, Sanchez MI, & Grichnik JM (2011). Molecular nevogenesis. Dermatology Research and Practice, 2011, 463184. 21754924 10.1155/2011/463184 PMC3130972

[R33] Sakaizawa K, Ashida A, Uchiyama A, Ito T, Fujisawa Y, Ogata D, … Uhara H (2015). Clinical characteristics associated with BRAF, NRAS and KIT mutations in Japanese melanoma patients. Journal of Dermatological Science, 80, 33–37. 26282084 10.1016/j.jdermsci.2015.07.012

[R34] Schalka S, Dos Reis VM, & Cuce LC (2009). The influence of the amount of sunscreen applied and its sun protection factor (SPF): Evaluation of two sunscreens including the same ingredients at different concentrations. Photodermatology, Photoimmunology & Photomedicine, 25, 175–180. 10.1111/j.1600-0781.2009.00408.x 19614894

[R35] Seite S, Fourtanier A, Moyal D, & Young AR (2010). Photodamage to human skin by suberythemal exposure to solar ultraviolet radiation can be attenuated by sunscreens: A review. The British Journal of Dermatology, 163, 903–914. 20977441 10.1111/j.1365-2133.2010.10018.x

[R36] Shain AH, Yeh I, Kovalyshyn I, Sriharan A, Talevich E, Gagnon A, … Bastian BC (2015). The genetic evolution of melanoma from precursor lesions. The New England Journal of Medicine, 373, 1926–1936. 26559571 10.1056/NEJMoa1502583

[R37] Thomas NE, Edmiston SN, Alexander A, Groben PA, Parrish E, Kricker A, … Conway K (2015). Association between NRAS and BRAF mutational status and melanoma-specific survival among patients with higher-risk primary melanoma. JAMA Oncology, 1, 359–368. 26146664 10.1001/jamaoncol.2015.0493 PMC4486299

[R38] Viros A, Pederson M, Furney SJ, Girotti M, Saturno G, Galvani E, … Marais R (2016). Abstract 4167: Ultraviolet radiation cooperates with individual oncogenes to drive melanomagenesis through distinct molecular mechanisms. Cancer Research, 76, 4167.

[R39] Viros A, Sanchez-Laorden B, Pedersen M, Furney SJ, Rae J, Hogan K, … Marais R (2014). Ultraviolet radiation accelerates BRAF-driven melanomagenesis by targeting TP53. Nature, 511, 478–482. 24919155 10.1038/nature13298 PMC4112218

[R40] Whiteman DC, Whiteman CA, & Green AC (2001). Childhood sun exposure as a risk factor for melanoma: A systematic review of epidemiologic studies. Cancer Causes & Control, 12, 69–82. 11227927 10.1023/a:1008980919928

[R41] Yaman B, Akalin T, & Kandiloglu G (2015). Clinicopathological characteristics and mutation profiling in primary cutaneous melanoma. The American Journal of Dermatopathology, 37, 389–397. 25357015 10.1097/DAD.0000000000000241

[R42] Zaidi MR, Davis S, Noonan FP, Graff-Cherry C, Hawley TS, Walker RL, … Merlino G (2011). Interferon-gamma links ultraviolet radiation to melanomagenesis in mice. Nature, 469, 548–553. 21248750 10.1038/nature09666 PMC3140101

