# Supplementary material for: Ultraviolet radiation accelerates NRas-mutant melanomagenesis: A cooperative effect blocked by sunscreen
Source: Pigment Cell Melanoma Res. Author manuscript; Available in PMC 2026 Jun 17. (PMC13273516; doi:10.1111/pcmr.12601)
Supplement: legends [file NIHMS2180339-supplement-legends.docx]

**Supplementary Figure 1** *TN^61R^*-UV tumors show evidence of *p16* loss and/or p53 stabilization. (a) Each circle represents 18s normalized p16^INK4a^ mRNA expression from a single tumor as determined by real-time RT-PCR. *TpN^61R^* tumors were used as a control for loss of *p16^INK4a^* expression. Of the 11 *TN^61R^* samples, only the sample shown in red lacks p16^INK4a^ expression. (b) Each circle represents the average percentage of p53-positive cells in a given tumor as determined by inForm assessment of five independent fields. p53 high tumors are those within the top 33 percentile. The closed blue triangle represents p53 positivity in a skin sample taken six hours post-UV (positive control). p-values were calculated using unpaired two-tailed t-tests with Welch's correction. Images representative of high, medium, and low staining are shown below.

**Supplementary Figure 2** Levels of individual active ingredients do not impact the melanoma preventative efficacy of commercial sunscreens. (a-e) To determine relative levels of each active ingredient applied, the amount of active ingredient in each product was multiplied by the sunscreen's relative coverage (See Table 1). Linear regression shows the correlation between levels of each applied component and median melanoma free survival (MFS). Gray lines represent 95% confidence intervals.

**Supplementary Figure 3** Sunscreens block cyclobutane pyrimidine dimer (CPD) formation caused by UV exposure. (a) Time course of CPD staining intensity as determined by inForm Software analysis in total skin nuclei taken from wild type mice after UV exposure. Data are mean ± standard deviation representing four to five mice per time point with five fields of view from each mouse. (b) Representative images of immunohistochemical staining for CPDs in sunscreen treated *TpN^61R^* skins six hours post-UV. NS = No Sunscreen, numbers correspond to individual sunscreen products.

**Supplementary Figure 4** Aerosol sunscreens exhibit variable coverage dependent upon lot (a) and product usage (b). Data represent the mean and standard deviation of four replicates performed on separate occasions. p-value calculated versus ‘Lot 1’ or ‘Off the shelf’ for each sunscreen using unpaired two-tailed t-tests with Welch’s correction. * = p ≤ 0.05, ‡ = p ≤ 0.01
